# Mechanical loading, an important factor in the evaluation of ion release from bone augmentation materials

**DOI:** 10.1038/s41598-018-32325-1

**Published:** 2018-09-21

**Authors:** Kathleen MacDonald, Daniel Boyd

**Affiliations:** 1 School of Biomedical Engineering, Dalhousie University, Halifax, B3H 4R2 Canada; 2 Applied Oral Sciences, Dalhousie University, Halifax, B3H 4R2 Canada

## Abstract

The controlled release of therapeutic inorganic ions from biomaterials is an emerging area of international research. One of the foci for this research is the development of materials, which spatially and temporally modulate therapeutic release, via controlled degradation in the intended physiological environment. Crucially however, our understanding of the release kinetics for such systems remains limited, particularly with respect to the influence of physiological loading. Consequently, this study was designed to investigate the effect of dynamic mechanical loading on a composite material intended to stabilize, reinforce and strengthen vertebral bodies. The composite material contains a borate glass engineered to release strontium as a therapeutic inorganic ion at clinically relevant levels over extended time periods. It was observed that both cyclic (6 MPa 2 Hz) and static (4.3 MPa) compressive loading significantly increased the release of strontium ions in comparison to the static unloaded case. The observed alterations in ion release kinetics suggest that the mechanical loading of the implantation environment should be considered when evaluating the ion release kinetics.

## Introduction

Percutaneous vertebroplasty (PVP), is a minimally invasive technique through which bone cement is injected directly into a fractured vertebral body, providing mechanical support and immobilization without the need for invasive orthopedic surgery. PVP provides rapid fracture fixation for osteoporotic vertebral compression fractures, resulting in rapid pain relief for 88% of patients^1^. Despite the positive impact that PVP has on the quality of life for patients, subsequent fractures (in adjacent vertebrae), subsequent to the original, are frequently associated with procedural outcomes. While the etiology of adjacent fractures is complex, there is consensus in the literature that principal contributory factors, are compromised bone health (e.g. osteoporosis), and anatomical misalignment(s) caused by kyphotic deformity (a forward leaning arch of the spinal column)^2^. To address these factors, prophylactic augmentation of adjacent vertebrae has been proposed, and demonstrated, to increase the ability to resist loads which would cause fracture^3^; thereby providing a means to mitigate subsequent fracture rates. Furthermore, as subsequent fractures have been linked to the progression of osteoporosis, the inclusion of therapeutically active agents, capable of slowing the progression of osteoporosis is considered to add further benefits for patients undergoing PVP whilst decreasing fracture rate and improving quality of life.

One approach to achieving a therapeutically active biomaterial for PVP has been associated with the inclusion of strontium ions (Sr^2+^), a therapeutically active inorganic ion, into PVP bone cements. Several clinical studies have confirmed the beneficial effects of pharmacologically active Sr^2+^ on bone quality^4–8^. The concomitant mechanism of action associated with Sr^2+^ is related to its ability to act as both (i) an antiresorptive and (ii) an anabolic agent on bone metabolism^9–11^. However, while Sr^2+^ has beneficial effects on bone quality, through direct action on osteoblasts and osteoclast cell lines^12^, its release levels must be controlled to ensure temporal maintenance of Sr^2+^ levels within the therapeutic window. While a variety of approaches have been used to incorporate Sr into orthopedic biomaterials^13,14^ little work exists with respect to investigating such devices as drug releasing systems (i.e. therapeutic ion release systems); particularly with respect to investigating the release kinetics and temporal maintenance of therapeutic thresholds^15^.

For composite resin cements, intended for both dental and orthopedic applications, hydrophilic modifications have been used to alter ion release kinetics. Such modifications increase water sorption and therefore degradation of the inorganic filler phase; frequently a bioactive glass^16,17^. As filler degradation, and ion release are dependent on the movement of water through the system, degradation constrained by hydrostatic forces. Furthermore, the rate of glass dissolution has been shown to vary greatly depending on solution effects, slowing rapidly in constrained volumes when degradation products can accumulate at the glass-solution interface^18^. Due to the complexity of the implanted *in vivo* environment, and the ethical restrictions on the use of animal models, simplified *in vitro* elution models are frequently used to assess drug release. In particular, changes to the elution media (including ionic concentration and organic molecule interaction), along with flow through conditions have been explored to better understand those factors which govern *in vivo* release kinetics^19,20^.

However, the effects of dynamic loading on therapeutic ion release, has received little attention by comparison. This gap in our knowledge is significant, especially given the emergence of research pertaining to metals in medicine, and in particular with respect to the temporal modulation of therapeutic metal ion release from biomaterials^21,22^. In drug-releasing hydrogel materials, cyclic compressive loading has been demonstrated to significantly increase the rate release of reversibly bound drug particles^23^. This effect is attributed to variations in hydrostatic pressure, creating a periodic reversal of water flow in and out of the material. During unloaded phases, hydrophilic forces draw unsaturated fluid into the material generating swelling forces. In this unsaturated environment, water-soluble drugs are rapidly released into solution. Conversely, during the compressive loading phase compressive strain drives fluid from the material voids, into the bulk solution, which is replaced with unsaturated fluid in the next unloaded phase continuing the cycle. While vertebroplasty materials are much less compliant than hydrogel materials, the addition of small hydrophilic mono-functional monomers, such as poly- hydroxyl ethyl methacrylate (HEMA) to composite resins has been shown to not only alter water sorption behavior but also to reduce the modulus of elasticity of composite resins by up to 40%^24^. As such hydrophilic modifications of composite resin cements may result in loading dependent alterations of bioactive glass filler degradation kinetics. Accordingly, to design a Sr^2+^ releasing bone cement which can provide appropriate *in vivo* levels of ion release, the ion release kinetics under mechanically loaded conditions must also be fully considered.

This study aims to examine how dynamic loading of hydrophilic composite resins alters the movement of water into and out of the system, while also providing insights into the mechanisms of Sr^2+^ release from such materials. The methods and data generated in this paper may further help to evaluate and regulate the performance of ion releasing medical devices in the future. Accordingly, and to achieve the research objective, a highly degradable borate glass was selected as the reinforcing phase for a resin composite bone cement comprising conventional constituent polymers. The glass phase selected for this work has been previously studied, and is known to provide for Fickian controlled diffusion of Sr^2+^ up to 60 days^25^. A highly hydrophilic Bis glycydil dimethacrylate (Bis GMA), HEMA polymer blend was chosen as the resin phase of the composite; with the particular ratios of glass and HEMA content being based on previous investigations by MacDonald *et al*. to maximize ion release efficiency over 60 days^15^. Cement specimens produced in this study were exposed to three incubation conditions while immersed in simulated body fluid (SBF); (i) cyclic compressive loading, (ii) static loading, and (iii) an unloaded control condition. Ion release into solution was assessed using inductively coupled plasma optical emission spectroscopy (ICP-OES).

## Results

**Figure 1:**
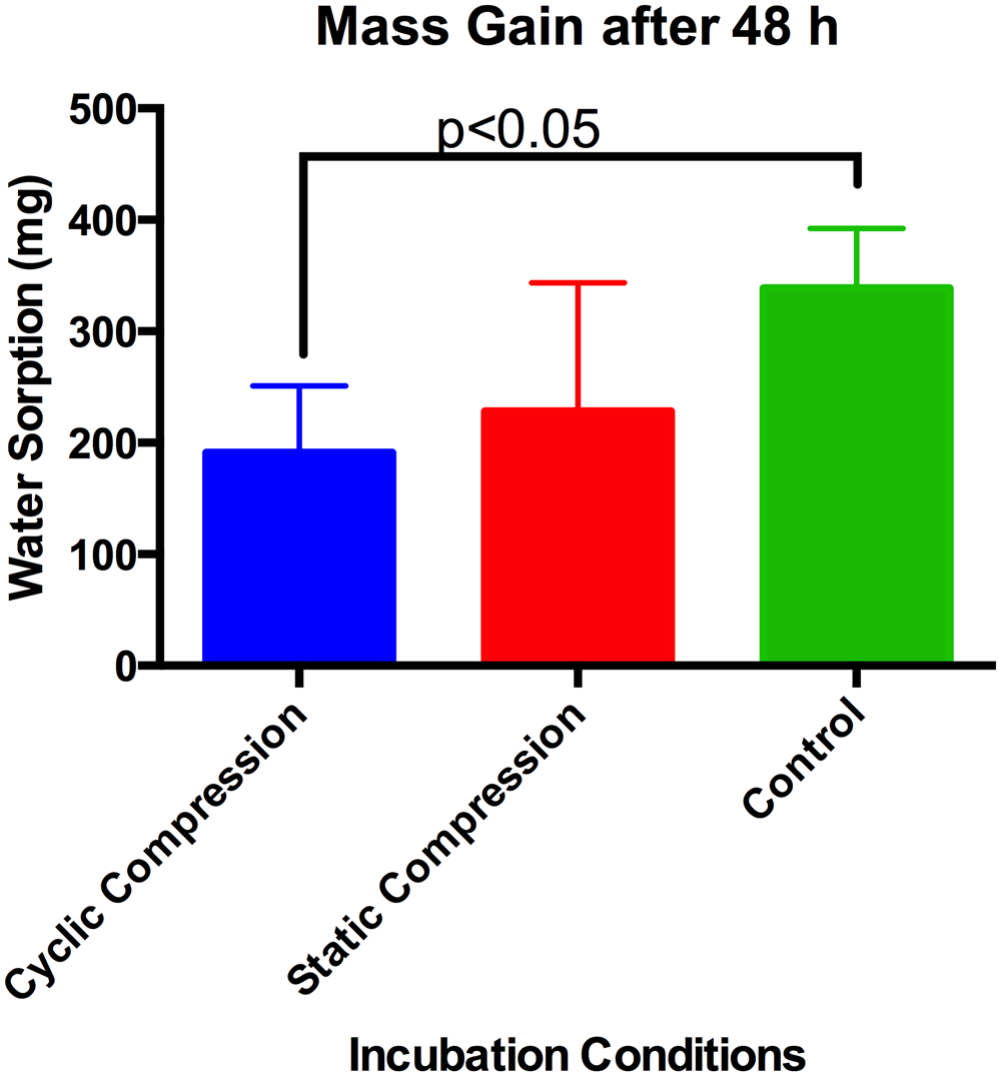
Mass gain (measured as difference between dry and wet weight) during cement incubation under various loading conditions, denoting statistically significant difference between unloaded and cyclic compression conditions (mean values with error bars representing standard deviations presented).

**Figure 2:**
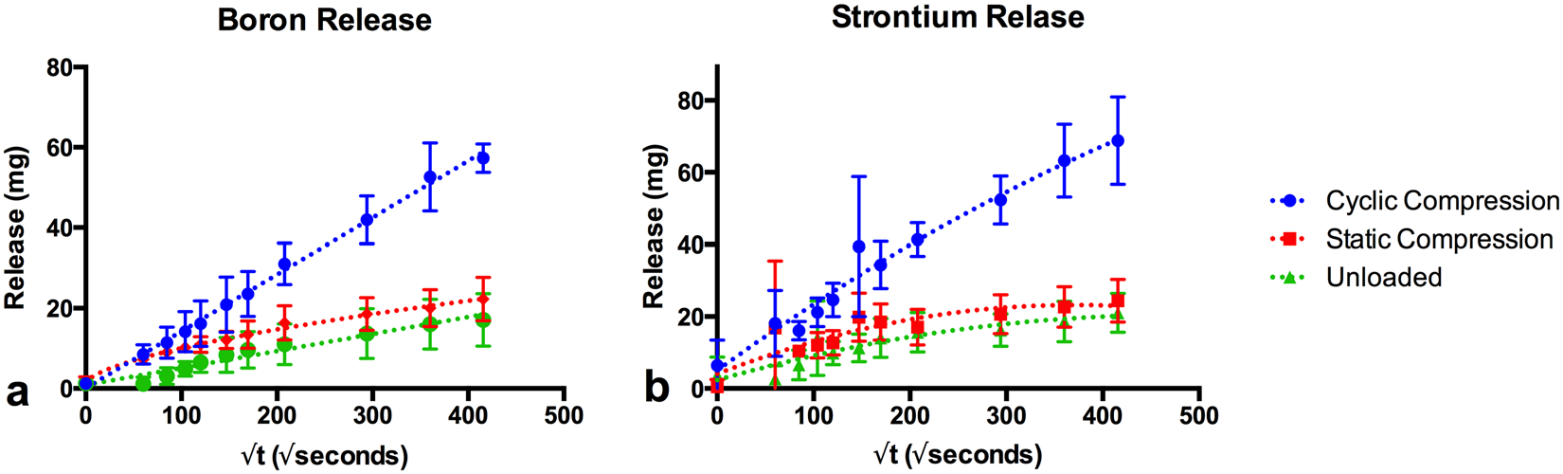
Cumulative release of (**a**) boron, and (**b**) strontium ions under various loading conditions, demonstrating Fickian diffusion under cyclic loading, and less Fickian diffusion in unloaded and static compression loaded incubation (mean values with error bars representing standard deviations presented).

Water sorption by cement samples was significantly decreased during cyclic loading (p < 0.05), with no decreases in water sorption being observed under static compression relative to unloaded samples (Fig. 1). The highest ion release levels were seen under cyclic compression loading, with the cumulative release of Sr and B reaching 68 and 57 mg respectively at 48 hours (Fig. 2). Cumulative release under static compression reached maximums of 24 and 22 mg for Sr and B respectively, while unloaded conditions achieved a maximum of 21 and 17 mg respectively. A significant increase in Sr release was seen in both the static (p < 0.05) and cyclic compression (p < 0.01) loading conditions relative to the unloaded control samples (Table 1), with the cyclic loading showing the greatest effect on cumulative ion release levels. While significant differences between static compression and unloaded samples at individual time points was observed only in the 1 and 6 hour samplings, an overall significant group effect (p < 0.001) was observed between each loading condition matching.

**Table 1:** Two-way ANOVA P values comparing strontium release between loading conditions at each time point.

Time (h)	Cyclic Compression	Cyclic Compression	Static Compression
	Static Compression	Unloaded	Unloaded
1	N.S.	<0.01	<0.01
2	N.S.	<0.01	N.S.
3	<0.01	<0.05	N.S.
4	<0.01	<0.01	N.S.
6	<0.01	<0.01	<0.05
8	<0.01	<0.01	N.S.
12	<0.01	<0.01	N.S.
24	<0.01	<0.01	N.S.
36	<0.01	<0.01	N.S.
48	<0.01	<0.01	N.S.

**Figure 3:**
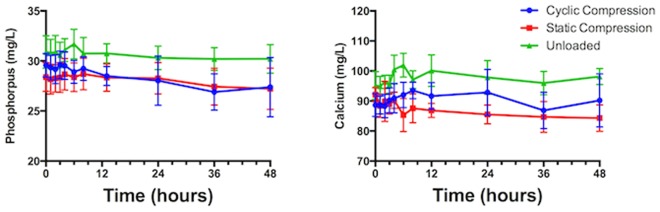
Calcium and Phosphorous concentrations in SBF over 48 hours in various loading conditions.

Akaikes informative criterion comparison of surface controlled, and diffusion controlled release models, preferred the more complex power series diffusion controlled release for all data sets (Table 2). When fitted to a modified Korsemyers Peppas model (with allowance for burst release effect^26^) power coefficients were (i) 0.499, 0.1835 and 0.360 for the release of Sr and (ii) 0.510, 0.306, 0.440 for the release of B under cyclic compression, static compression, and unloaded conditions respectively. While no significant difference was observed in the ratio of Sr to B released (supplemental Fig. 1) between loading conditions, a significant decrease (p < 0.01) was observed between early (1–3 h) time points and late (36–48 h) time points for the unloaded samples only. No significant changes were observed in the calcium concentration of the SBF over the 48 h experiment (Fig. 3). Significant decreases were observed for the 24, 36, and 48 h time points under cyclic compression, and for the 48 hour time point under static compression (Fig. 3).

**Table 2:** Comparison of fits of linear release model (surface controlled release) and power series release with allowance for burst effect (with X denoting time in seconds, and Y denoting cumulative release in mg, model variables B and C denote the coefficient of time dependence, and burst release respectively).

Comparison of Fits		Cyclic Compression	Static Compression	Unloaded
Simpler model		First order polynomial (straight line)	First order polynomial (straight line)	First order polynomial (straight line)
Probability it is correct		<0.01%	<0.01%	<0.01%
Alternative model		Power series: Y = A*X^B^ + C	Power series: Y = A*X^B^ + C	Power series: Y = A*X^B^ + C
Probability it is correct		>99.99%	>99.99%	>99.99%
**Ratio of probabilities**				
Preferred model		Power series: Y = A*X^B^ + C	Power series: Y = A*X^B^ + C	Power series: Y = A*X^B^ + C
Difference in AICc		46.64	29.51	18.78
Time Coefficient of Release (B)	Sr	0.50	0.18	0.36
	B	0.51	0.31	0.44

## Discussion

**Figure 4:**
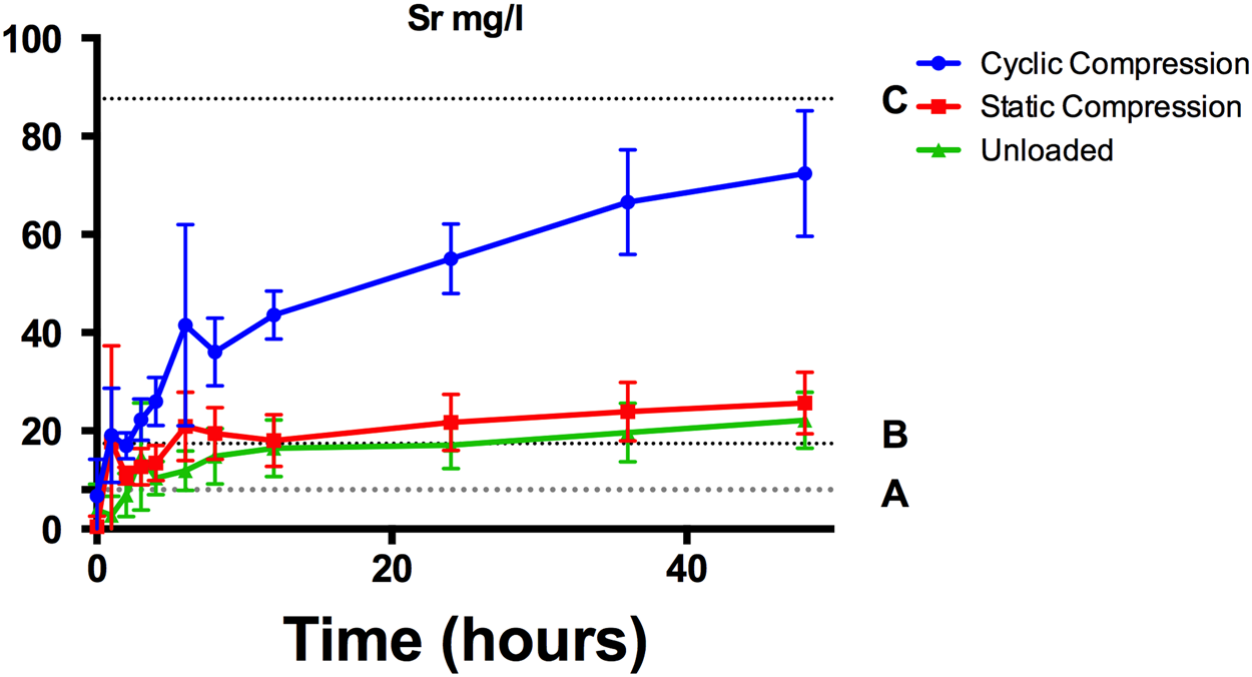
Sr release from cements into SBF in mg/L, relative to (**A**) *in vitro* osteoblast activation threshold^10^ (**B**) co-cultured osteoclast inhibition levels^28^ and (**C**) *in vitro* osteoclast inhibition threshold^52^ (mean values with error bars representing standard deviations presented).

Sr release (24–48 mg/L after 48 h) was much higher than previous studies of a similar material in phosphate buffered saline^15^. Elution studies of a 45% HEMA, 26.5% Bis GMA, 26.5% triethylene glycol dimethacrylate (added as a diluent to reduce viscosity) reported strontium release levels below 4 mg/L after 24 hours, and below 20 mg/L after 60 days^15^. Release of B however was similar to previously reported values of 16 mg/L at 24 hours in both unloaded and statically compressed samples. These results suggest that the ionic composition of the elution media is of greater consequence for sparingly soluble elements such as Sr. Cumulative strontium release remained above the *in vitro* efficacy levels for osteoblast proliferation, but below *in vitro* osteoclast inhibitory levels (Fig. 4)^9,10,27^. While high levels of strontium are required to inhibit osteoclasts in monoculture, systemic therapy has proven to provide effective anti catabolic effects at serum levels as low as 10 mg/L^27^. This lowered threshold may be due to the osteoblast mediated decrease in osteoclast activity, as previously demonstrated by Tat *et al*., in which the threshold for decreased bone resorption was decreased from 174.4 mg/L with monoculture osteoclasts to 17.4 mg/L when co-cultures of osteoblasts and osteoclast precursors were investigated^28^. The serum Sr levels for co-cultured decreased resorption were reached after 24 hours under unloaded conditions, or 3 hours under cyclic loading demonstrating that the potential for anti-osteoporotic effect is increased under mechanical loading. Much greater overall release, under the cyclic loading condition, suggests that the static incubation conditions widely used for the investigation of strontium releasing bone cements will underestimate the true release levels and therefore impact effective local dosing. While these results highlight the importance of appropriate mechanical modeling of the implantation environment, appropriate selection of ratio of cement to SBF used would be needed to accurately predict Sr release *in vitro*. While studies focusing on the formation of apatite on the surface of bioactive glasses have suggested mass normalized incubation conditions with much lower glass to volume ratio than used in this study^29^, the surface to volume ratio utilized was constrained to 15 ml/cm^2^ due to the geometry of the mechanical testing environment used.

While cyclic loading increased therapeutic Sr release, it also increased the release of B raising a potential concern for cytotoxicity. Levels of boron exposure as low as 1.3 mM (14.5 ppm) have previously been associated with both decreased ALP activity, and osteoclast differentiation in *in vitro* conditions^30^. The use of dynamic incubation conditions is noted to have a strong effect on the cytotoxicity of borate bioactive glasses under *in vitro* conditions, with observed inhibition levels below expected inhibition based on ion release^31^. As such, no toxic effect has been observed in the direct culturing of MLO A5 cells on borate glass scaffolds despite expected boron concentration of up to 18 mM (194.4 mg/L)^32,33^. Similarly, animal implantation models have demonstrated favorable bone response to high borate glass materials^34–37^. While potential cytotoxic effect may be expected with the 58 mg release observed under *in vitro* testing conditions used in this study, further evaluation would be needed to assess the effect of boron release on cement biocompatibility.

While previous studies have reported a t^1/2^ dependence for ion release from bioactive glass reinforced composite resin dental materials^38^, such kinetics were only seen in the cyclic loading conditions of this study. In the static compression and unloaded conditions the power model coefficients were below 0.5, characteristic of “less Fickian” release^39^. This finding suggests that the polymer chain relaxation rate under the unloaded, and static compressive conditions is much faster than the rate of water penetration. This increased rate of relaxation is likely an effect of the high mono-functional HEMA content, decreasing the fraction of bi-functional monomers reducing crosslinking. Previous studies into the release of gentamycin from Poly methyl methacrylate bone cements reported crack surface mediated increases in drug release rates. A study of antibiotic loaded cements under uniaxial sinusoidal tension-compression (±10 MPa at a frequency of 2 Hz) resulted in a three-fold increase in the rate of gentamicin released versus statically incubated samples; with both conditions displaying surface controlled release^40^. It was proposed that the increased rate of release was due to crack formation providing an increased surface area for drug elution. When a 400 KPa 5 Hz compressive force was applied to the stem of a cemented hip prosthesis in a model elution environment (cement stresses of −2 MPa to 6 MPa) minor increases in antibiotic elution were seen only in a highly porous cement formulation over 28 days of elution testing but no alterations to the mechanism of release were reported^41^. While crack surface mediated release mechanisms are considered in the literature, such a mechanism is unlikely to explain the results seen in this study, as any increase in surface area would result in *increased* water sorption.

Results from this study, in contrast, indicate significant alterations to the mechanism of release of Sr^2+^ as a result of physiological loading variations. Increases to power law release coefficients suggest that compressive loading decreased both the rate of water sorption and polymer chain relaxation in both cyclic and static conditions. Mechanical loading of hydrogels has been demonstrated to alter release kinetics only for *reversibly bound* growth factors. Such an effect may explain the alteration in kinetics seen in this study. Polar pendant groups in hydrophilic poly HEMA materials are associated with cation chelation in their hydrated state^42^, providing both a mechanism of hydrogel calcification and bone integration. While cyclic loading alters local concentration gradients, allowing for increased ion release, compressive loading also resulted in slower chain relaxation, decreased pendant group exposure, and potentially decreased cation chelation. Compressive loading however also resulted in mineralization as evidenced by a significant decrease in phosphorous content in solution for both cyclic and static compression loading, (no evidence of mineralization was observed for the unloaded condition). While the decrease in the ratio of strontium to boron released from the unloaded sample may be evidence of retention of strontium within the cement sample, no decrease in phosphate was observed suggesting this effect was not likely to be the result of strontium phosphate formation. The mechanism underlying the increased rate of ion release under static compressive loading, despite decreased water sorption, remains unclear. Due to the use of silica free glass in this study salinization was not performed, and the filler was thus uncoupled, potentially resulting in increased surface filler loss^43^. The increased rates of water sorption and chain relaxation observed in the unloaded specimens would suggest increased matrix swelling, which may have served to retain a higher fraction of surface particles, and contributed to the lower rates of ion release in these samples. Imaging of the hydrated resin surface would be required to verify if changes to filler loss occurred between loading conditions. Alternatively, further investigation into the mineral phase formed, and if it is located (1) at the glass surface due to altered glass degradation, (2) at the resin surface due to changes in the relaxation rate of the polymer, or (3) within the bulk of the solution due to saturation effects, would be needed to better understand this phenomena. Surface analysis through XRD, or SEM would be of utility to provide further insight into how compressive loading results in increased release.

While continuous cyclic loading is standard in material testing, *in vivo* loading would present fewer, intermittent loading cycles, with ASTM F2118 standards for cyclic testing of acrylic bone cement reporting an average of 2–3 million steps a year^44^. As such the 7,200 loading cycles per hour used in this study would approximate a full day of activity, with the 48 h duration approximating 48 days of activity. While this is a short duration study, this may indicate a potential mechanism to extend the effective window of testing.

The loading levels selected in this study were based on computational models assessing the cement stress levels during walking following vertebral body augmentation, assuming fill volumes of *ca*. 8 ml^45^. True loading values may vary significantly due to the variability in fill pattern and irregularly shaped cement volume seen following vertebroplasty^46^. Previous models have predicted cement stress as low as 1.5 MPa within the cement bolus (based on an assumption of 70% fill)^47^. Furthermore the elastic modulus of trabecular bone can vary by orders of magnitude in even healthy individuals, adding variability to the extent of stress shielding, while kyphotic angle has been reported in increased quasi-static compressive forces by as much as 2–3% per degree of kyphosis^48,49^. As such our limitations in predicting the loading levels in augmented vertebrae limits our ability to predict cement behavior, including ion release kinetics. Similarly, it is important to note that the implantation environment is temporally heterogeneous, and would be expected to change over the course of healing. Specifically, changes in blood flow, local cellular activity, and load distributions would be expected to evolve as the fracture heals. While the results of this study demonstrate the ability of cyclic loading conditions to alter ion release kinetics over a short period of investigation, further investigation into the threshold of this effect, as well as long-term release behavior is merited. Along with variations in loading conditions, alterations to solution media, notably the inclusion of serum proteins would serve to better mimic the true implantation environment deepening our understanding of material host interactions which mediate ion release.

## Conclusion

Both cyclic and static compressive stress has been demonstrated to shown to yield a significant increase in ion release from a borate glass filled composite resins (relative to unloaded release conditions). Cyclic loading conditions may provide a mechanism of extending the time frame for diffusion-controlled release of therapeutic inorganic ions from such systems, despite the decrease in water sorption caused by compression. The study results indicate that the mechanical loading environment should be considered when assessing the release kinetics of a drug loaded bone augmentation material for both toxic and therapeutic effects.

## Methods

A two paste chemically cured resin blend was fabricated from a blend of Bis Glycydil Dimethacrylate (Bis-GMA) and Hydroxyl Ethyl Methacrylate (HEMA) based on the compositions in Table 3, mixed in the appropriate weight ratios with either benzyl peroxide (BPO), or 2,2′-(4-Methylphenylimino) diethanol (DHEPT), to initiate the free radical polymerisation reaction upon mixing. Resin blends were shielded from light and mixed in a shaking incubator at 37 °C, 2 Hz overnight to achieve homogeneous mixture. The borate-glass reinforcing phase was fabricated as described by MacDonald *et al*.^25^, ground and sieved to retrieve sub 45 micron particles. Each cement paste was formed from a mixture of glass filler (60 wt %) mixed to homogeneity with either resin part one or two (40 wt %), through hand spatulation. Cement cylinders were fabricated using a cylindrical aluminum split mold (4 cm diameter, 3 cm height) filled with an equal mixture of the two pastes (40 g/paste, 80 g total) through hand spatulation, clamped between two stainless steel plates, and allowed to cure for and hour. Samples were removed from the mold, and stored in a desiccator overnight until the elution study.

**Table 3:** Two paste chemically cured resin system, by weight percentage.

Component	Paste 1	Paste 2
Bis GMA	54%	53%
HEMA	45%	45%
BPO	1%	0%
DHEPT	0%	2%

### Incubation conditions

Cylindrical cement samples (n = 4 per loading condition) were incubated at 37 °C in 950 ml (surface to volume ration of 15 ml/cm^2^) simulated body fluid^50^ for 48 hours under three loading conditions: (a) no mechanical loading, (b) cyclic compressive loading and (c) static compressive loading. Both cyclic and static compressive loading was performed using a Bose Electroforce 3510 system (TA Instruments, MN). Environmental control tank volume was reduced to approximately 1.5 L by the fixation of an acrylic cylinder (approximately 12.5 cm diameter, 25 cm height) to the chamber base centered around the loading platform using silicone marine adhesive on the outside rim. The outer tank was filled with water during testing to improve heat conduction from elements and stabilize temperature. Cyclic loading was performed as a compression-compression sinusoidal waveform, oscillating between 0.3 to 6 MPa at a frequency of 2 Hz. Static compressive loading was performed at 4.3 MPa (the equivalent RMS value of the cyclic loading conditions). Incubation media was sampled through the removal of 5 ml of SBF at 1, 2, 3, 5, 6, 8, 12, 24, 36 and 48 h, and replaced with 5 ml of fresh simulated body fluid.

### Water sorption measurements

Cement cylinders were removed from solution and patted dry of excess SBF before the measurement of wet weight. Cylinders were then dried in a 45 °C oven until constant mass was achieved and dry weight was recorded (approximately 7 days). Water sorption was recorded as the difference between wet and dry weights.

### Ion release

Ion release profiles were assessed through the use of inductively coupled plasma optical emission spectroscopy, for the presence of phosphorous, calcium, boron and strontium in solution. Extract media was diluted in 2% HCl to 1:6, 1:6000, 1:100 and 1:1000 dilutions for the assessment of phosphorous, calcium, boron and strontium respectively. ICP-OES measurements were performed in triplicate, against a standard calibration curve, and corrected for the removal of simulated body fluid during sampling. Ion release profiles were fitted through least squares regression to diffusion controlled release models, with model parameters compared between elution conditions using a corrected Akaike’s Information Criterion^51^ using Prism 6 (GraphPad Software, Ca). Two way ANOVA with Turkey’s multiple comparison honesty test was performed comparing ion release between loading states and time points using Prism 6.

## Electronic supplementary material

Supplementary Figure

## Data Availability

The datasets generated during and/or analysed during the current study are available from the corresponding author on reasonable request.
