# Supplementary material for: Mechanical loading, an important factor in the evaluation of ion release from bone augmentation materials
Source: Sci Rep. 2018 Sep 21;8:14225. doi: 10.1038/s41598-018-32325-1 (PMC6154963; doi:10.1038/s41598-018-32325-1)
Supplement: Supplementary file 1 — Supplementary Figure [file 41598_2018_32325_MOESM1_ESM.docx]

**Mechanical loading, an important factor in the evaluation of ion release from bone augmentation materials**

**Kathleen MacDonald^1^, Daniel Boyd ^1,2*^**

^1^School of Biomedical Engineering, Dalhousie University, Halifax, B3H 4R2, Canada

^2^Applied Oral Sciences, Dalhousie University, Halifax, B3H 4R2, Canada

*Correspondence to d.boyd@dal.ca


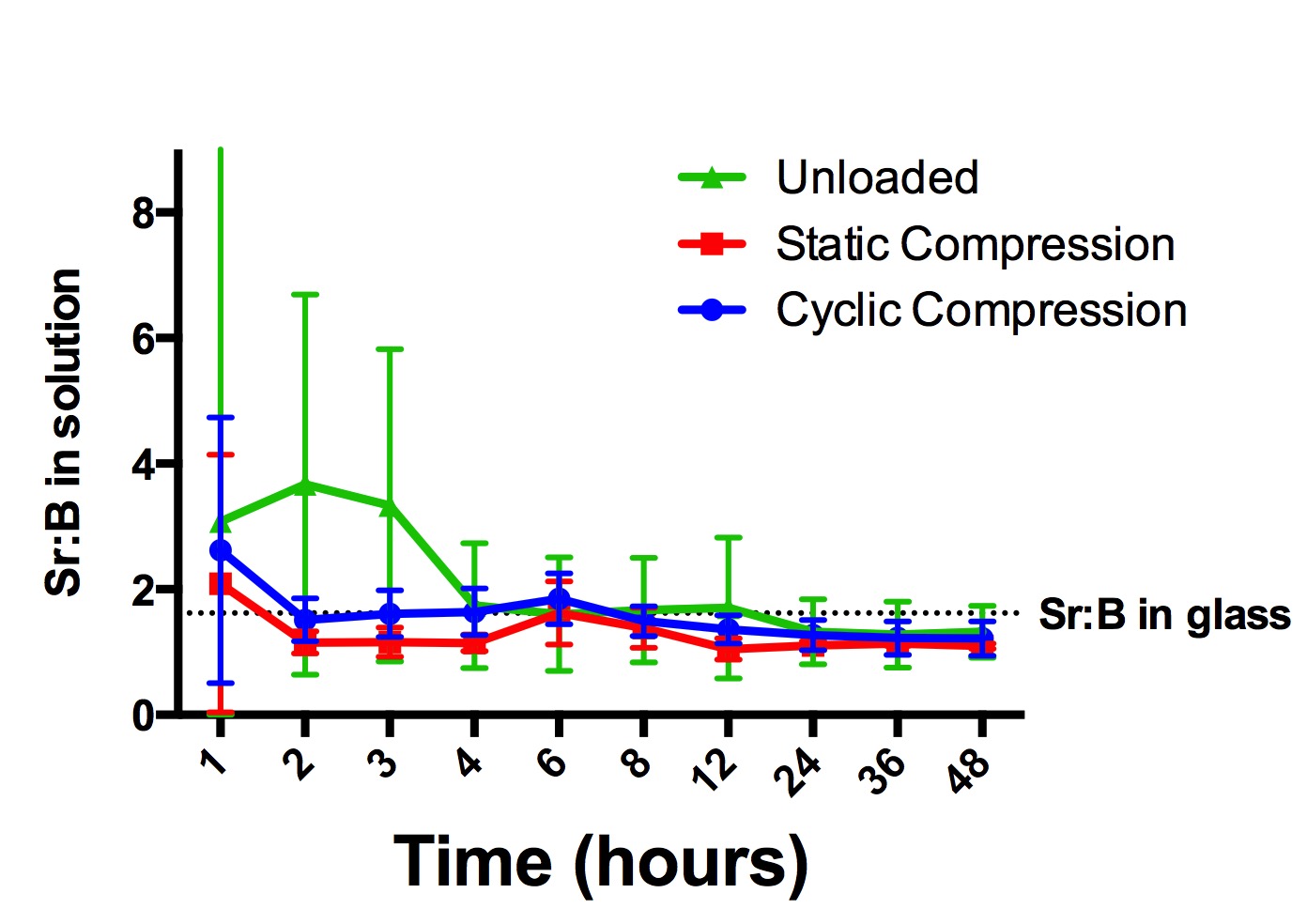


**Supplementary Figure S1:** Ratio of strontium to boron released from composite under varying loading conditions (on a mass/mass basis)
